# Generation of the *Krt24-Cre^ERT2^* Mouse Line Targeting Outer Bulge Hair Follicle Cells

**DOI:** 10.3390/ijms26073165

**Published:** 2025-03-29

**Authors:** Jiao Wang, Yifei Qiu, Yansheng Zhu, Xuejiao Ren, Xiaoqi Zhou, Xia Wang, Huiyang Song, Jianhao Li, Chengming Gao, Gangqiao Zhou, Pengbo Cao

**Affiliations:** 1 College of Life Science, Hebei University, Baoding 071002, China; wja0103@163.com (J.W.); 15164542322@139.com (X.Z.); 2 State Key Laboratory of Medical Proteomics, National Center for Protein Sciences at Beijing, Beijing Institute of Radiation Medicine, Beijing 100850, China; 3180101060@zju.edu.cn (Y.Q.); 17776836272@163.com (X.W.); a1291166857@163.com (H.S.); gchengming1988@163.com (C.G.); 3 College of Life Sciences, Anhui Medical University, Hefei 230032, China; yszhu810@163.com; 4 Collaborative Innovation Center for Personalized Cancer Medicine, Center for Global Health, School of Public Health, Nanjing Medical University, Nanjing 211166, China; renxjy@163.com; 5 Hengyang Medical College, University of South China, Hengyang 421001, China; 13986762047@163.com

**Keywords:** *Krt24*, outer bulge hair follicle stem cells, lineage tracing, ionizing irradiation

## Abstract

Outer bulge (OB) hair follicle stem cells (HFSCs) play a crucial role in maintaining hair follicle structural stability and regulating the hair follicle cycle. Previous studies demonstrated that keratin 24 (*Krt24*) exhibits spatiotemporally restricted expression in OB HFSCs. Here, we report the generation of the *Krt24-Cre^ERT2^* mouse line. When crossed with *Rosa26^LSL-tdTomato^* or *Rosa26^LSL-DTR^* reporter lines, offspring exhibited specific labeling (*Krt24-Cre^ERT2^;Rosa26^LSL-tdTomato^*) or ablation (*Krt24-Cre^ERT2^;Rosa26^LSL-DTR^*) of *Krt24^+^* cells. In *Krt24-Cre^ERT2^;Rosa26^LSL-tdTomato^* mice, phase-specific tamoxifen (TAM) administration demonstrated spatiotemporal fidelity of Cre activity to endogenous *Krt24* expression patterns. Lineage tracing revealed that tdTomato-labeled *Krt24^+^* cells differentiated into the outer root sheath (ORS) during the anagen phase and persisted when hair follicles reentered telogen. Ablation of *Krt24^+^* cells via diphtheria toxin (DT) administration significantly delayed anagen initiation. Mice under continuous depletion of *Krt24^+^* HFSCs experienced substantial mortality after ionizing irradiation. Notably, ionizing radiation triggered a marked expansion of tdTomato-labeled *Krt24^+^* cells, accompanied by maintained hair follicle homeostasis. Taken together, this study established a *Krt24-Cre^ERT2^* mouse line targeting OB HFSCs, which are essential for hair follicle development and damage repair.

## 1. Introduction

Hair follicle stem cells (HFSCs) play a crucial role in the formation of hair follicle (HF) structures and hair regeneration [1]. Within the narrow niche of the bulge and the hair germ (HG) during the telogen (resting phase), there are various molecularly labeled HFSCs that proliferate and differentiate into different structures of the HF during the anagen (growth phase) [2,3]. Outer bulge (OB) HFSCs, a monolayer of cells located on the outer layer of the HF bulge region and serve as the interface between the bulge and dermis [4]. During anagen, these cells expand downward and differentiate into the outer root sheath (ORS) while retaining precursor cells in the upper ORS region for subsequent HF cycles [1,5]. Notably, HFs lacking OB HFSCs exhibit significant developmental abnormalities and delayed HF cycling [6].

With the deepening investigation into the molecular mechanisms of HFSC activity and heterogeneity [5,7], there remains a critical need for robust evidence to dissect cellular functions within specific niches of the hair follicle. Keratin 24 (KRT24) is encoded by the *Krt24* gene, which is located at one end of the type I keratin gene cluster [8,9]. A primary characteristic of type I keratin family members is their expression in epithelial cells or epithelial appendages [8]. For example, members of this gene cluster, including *Krt25*, *Krt26*, *Krt27*, and *Krt28*, are expressed in the inner root sheath (IRS) of hair follicles [10]. Although *Krt14* and *Krt15* are broadly expressed in the hair follicle and interfollicular epidermis [11], they continue to serve as markers for epidermal basal stem cells or hair follicle stem cells in numerous studies [12,13,14,15].

*Krt24* initially garnered attention in hair follicle cycling because of its marked upregulation during the telogen phase, whereas other hair keratins exhibited minimal expression in telogen [16]. Subsequently, although studies have reported the spatiotemporal expression specificity of *Krt24* in hair follicles and suggested potential functional roles of *Krt24^+^* cell populations during epidermal injury repair, research on *Krt24* in hair follicles remains insufficient. Joost et al. highlighted in a single-cell transcriptomic study of epidermis and hair follicles that high *Krt24* expression characterizes OB HFSCs [17]. Gur-Cohen S. et al. utilized *Krt24* to localize bulge HFSCs in their investigation of how the hair follicle stem cell niche dynamically remodels to regulate tissue regeneration [18]. Beeler J.S. et al. employed *Krt24* as a marker of OB HFSCs to study their responses in tissue homeostasis and wound repair [19]. Adam R.C. et al. discovered that the transcription factor NFI plays a critical role in maintaining HFSC-mediated hair regeneration; NFI deficiency caused severe downregulation of stemness genes including *Krt24* and *Lgr5* in bulge HFSCs, with *Krt24* used to identify bulge HFSCs in this study [20]. Zhang C. et al. also applied *Krt24* to define OB HFSCs in hair follicle aging research [21]. In scRNA-seq studies of mouse skin, we observed the differential gene expression in OB HFSCs between the telogen and anagen phases [17,22]. In the telogen phase, OB HFSCs showed elevated expression of genes including keratin 24 (*Krt24*) [16], periostin (*Postn*), nuclear factor of activated T cells 1 (*Nfatc1*), etc. [5]. Conversely, these genes showed significant downregulation in the anagen phase. Notably, *Krt24* exhibited a unique expression profile compared with other HF keratins. Therefore, *Krt24* demonstrates spatiotemporal specificity for OB HFSCs in the telogen phase and can serve as a promising marker. Nevertheless, the field remains critically underequipped with genetic tools to interrogate the functional roles of *Krt24^+^* OB HFSCs in hair follicle cycling and cutaneous homeostasis.

In the field of HF research, mouse models are widely used for studying HF development and regeneration, as well as hair diseases, because of their convenient induction methods, stable cycles, and relatively simple skin structures. Because of the extreme complexity of HF microstructures and the strong heterogeneity of molecular expression profiles among cells [17,22], the Cre/Cre^ERT^-loxP recombinase system has become an essential tool for studying HF microenvironments. A series of Cre mice targeting pan-hair follicle stem cells (pan-HFSCs) or pan-epidermal stem cells have been generated, such as *Krt15-Cre^PR^*, *Krt14-Cre^ER^*, and *Krt5-Cre^ER^* [23,24,25]. Various signaling pathways influence HFSCs; consequently, pathway-specific Cre strains such as *Shh-Cre^ER^*, *Gli1-Cre^ER^*, and *Inv-Cre^ER^* were generated for studying Sonic hedgehog (*Shh*) signaling or Ras signaling [26,27,28]. Melanocyte stem cells (McSCs) play an important role in hair pigmentation, *Tyr-Cre^ER^* and *c-Kit-Cre^ER^* mouse lines have shown that oncogenically transformed McSCs are a significant source of melanoma [29,30].

Here, we generated the *Krt24-Cre^ERT2^* mouse line through targeted insertion of *Cre^ERT2^* into *Krt24* exon 8, employing zygotic coinjection of sgRNA and Cas9 mRNA for CRISPR editing. Crossing this mouse line with *Rosa26^LSL-tdTomato^* or *Rosa26^LSL-DTR^* mouse line generated offspring that could enable lineage tracing or conditional ablation of *Krt24^+^* cells. Lineage tracing after tamoxifen induction demonstrated that *Krt24^+^* OB HFSCs were predominantly localized in the telogen-phase hair follicle niche. Furthermore, conditional ablation of these cells significantly impaired hair follicle regeneration and reduced survival rates in mice following ionizing radiation injury, implying their pivotal role in tissue repair and stress response.

## 2. Results

### 2.1. Generation of the Krt24-Cre^ERT2^ Mouse Line Targeting Outer Bulge Hair Follicle Cells

**Figure 1:**
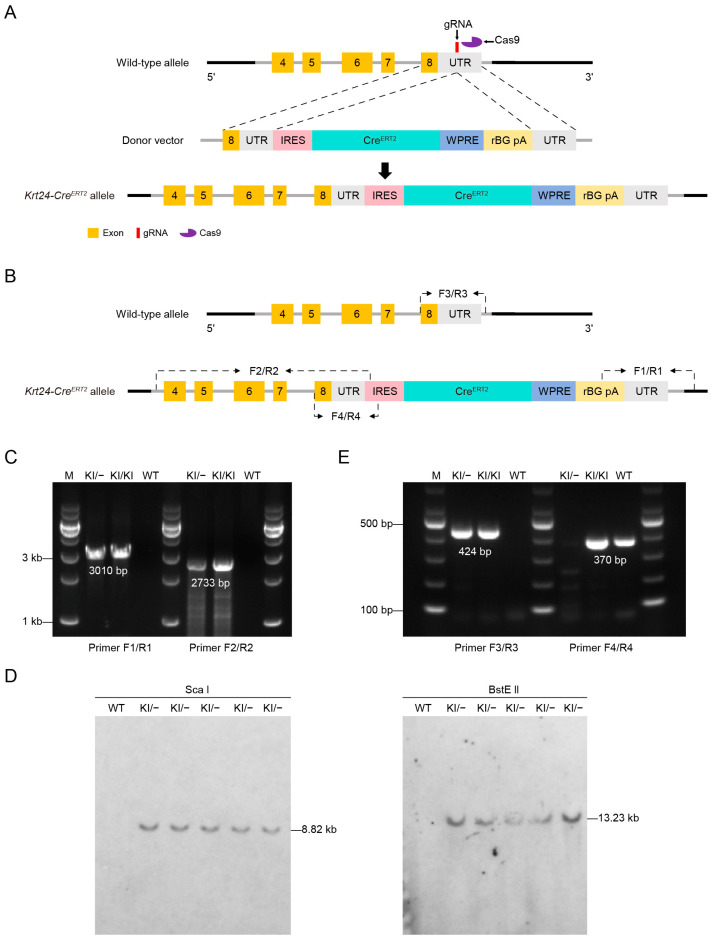
Generation of the *Krt24-Cre^ERT2^* mouse line targeting outer bulge hair follicle cells. (**A**) Schematic diagram illustrating the wild-type allele, donor vector, and targeting allele. The donor DNA contained the “IRES-Cre^ERT2^-WPRE-rBG-polyA” sequence. IRESs are RNA elements that recruit ribosomes to the internal region of mRNAs and initiate translation through a cap-independent pathway. The single guide RNA (gRNA) targeted the 3′ UTR region of mouse *Krt24* gene (5′-CACCAGACTGACCATTGTGCCGG-3′). (**B**) Schematic diagram showing the locations of the primers. (**C**) PCR products from primers F2/R2 and F1/R1 can span the 5′ and 3′ homology arms, respectively. The 3010 bp PCR product from primers F1/R1 and the 2733 bp product from primers F2/R2 were detected exclusively in KI/− and KI/KI mice, but not in WT mice. (**D**) Southern blot analyses of the *Krt24-Cre^ERT2^* knock-in allele. ScaI-digested genomic DNAs from wild-type and heterozygous (KI/−) mice were hybridized with the probe, and an 8.82 kb band from the targeted allele was detected. BstE II-digested genomic DNAs hybridized the probe, detecting a 13.23 kb band from the targeted allele. (**E**) PCR products of primers F3/R3 and F4/R4 could detect wild-type and knock-in alleles, respectively. The 370 bp product from the primer F4/R4 was observed in KI/− and KI/KI mice. The 424 bp product from the primer F3/R3 was observed in WT and KI/− mice, but not in KI/KI mice. The band labeled “M” represents the DNA marker. KI, knock in; WT, wild-type.

To specifically label *Krt24^+^* OB HFSCs, we generated *Krt24-Cre^ERT2^* mice, in which *Cre^ERT2^* was inserted into the 3′-UTR of *Krt24* locus (Figure 1A). The targeting vector contained an “IRES-Cre^ERT2^-WPRE-rBG-polyA” cassette. The internal ribosome entry site (IRES) allows the ribosome to initiate transcription from the 5′ end of the *Cre^ERT2^* gene directly and independently. Additionally, the woodchuck hepatitis virus posttranscriptional regulatory element (WPRE) significantly increases the expression level and translation efficiency of mRNA. The *Krt24-Cre^ERT2^* donor vector, sgRNA, and Cas9 mRNA were comicroinjected into mouse zygotes to generate offspring. *Krt24-Cre^ERT2^* offspring were identified by PCR using primers spanning the homology arms, confirming the accurate integration of the donor *Cre^ERT2^* DNA into the *Krt24* locus (Figure 1B). The primer pairs F1/R1 and F2/R2 were used for genotyping the wild-type (WT) and targeted allele, respectively (Figure 1C). Southern blot analysis using a probe against the transgenic sequence confirmed the accurate integration of the donor *Cre^ERT2^* DNA into the *Krt24* locus (Figure 1D). To optimize genotyping of the *Krt24-Cre^ERT2^* mice, primers F4/R4 were designed to amplify the inserted DNA, and primers F3/R3 were designed to amplify the wild-type allele (Figure 1E). During the breeding of *Krt24-Cre^ERT2^* mice, no embryonic lethality or abnormal breeding rates were observed (Supplementary Figure S1A,B), indicating the stability and normal reproductive capacity of this mouse line. Additionally, the insertion of *Cre^ERT2^* did not affect the expression of endogenous *Krt24* (Supplementary Figure S1C).

### 2.2. TdTomato-Labeled Krt24^+^ Cells Are Located in the Outer Bulge Region and Persist into the Next Hair Follicle Development Cycle

**Figure 2:**
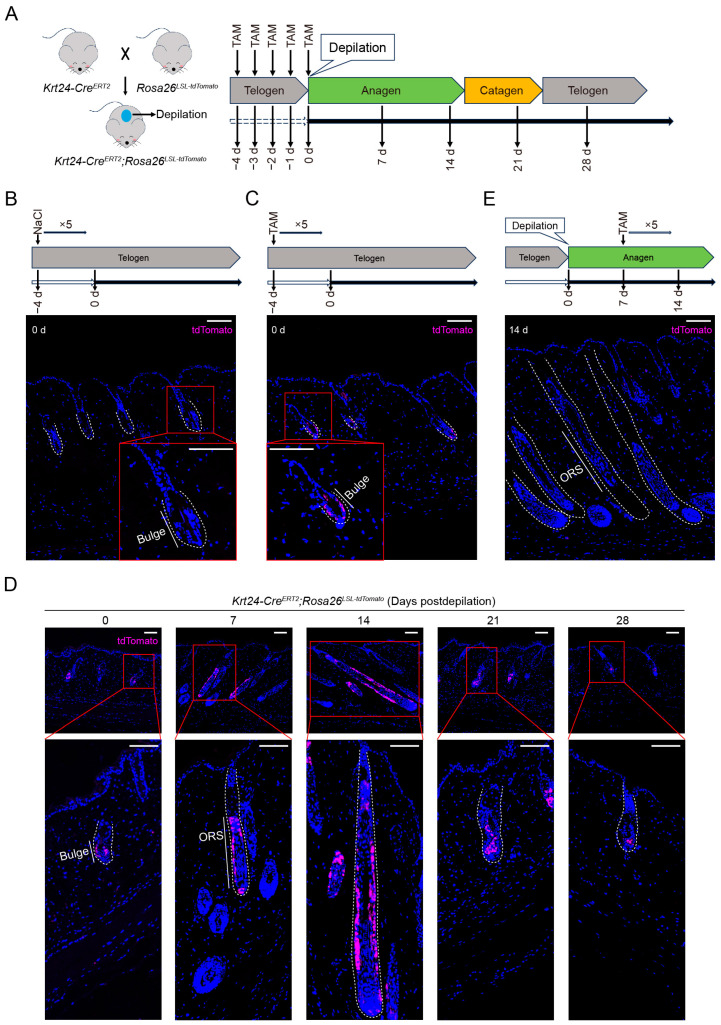
TdTomato-labeled *Krt24^+^* cells are located in the outer bulge region and persist into the next hair follicle development cycle. (**A**) The protocol demonstrates a strategy for tracking *Krt24^+^* cells and their progeny in *Krt24-Cre^ERT2^*;*Rosa26^LSL-tdTomato^* mice. Mice received intraperitoneal injections of TAM for 5 consecutive days, followed by depilation to induce an anagen phase transition in the hair follicles. Dorsal skin samples were collected at 0, 7, 14, 21, and 28 days postdepilation for immunofluorescence staining assays. (**B**) After *Krt24-Cre^ERT2^*;*Rosa26^LSL-tdTomato^* mice were treated by 0.9% NaCl for 5 consecutive days, no positive signal of tdTomato was detected in the dorsal skin after 2 weeks. *n* = 3 mice. (**C**) After treating *Krt24-Cre^ERT2^*;*Rosa26^LSL-tdTomato^* mice with TAM for 5 consecutive days, a tdTomato positive signal was detected in the hair follicle bulge after 2 weeks. *n* = 3 mice. (**D**) Immunofluorescence staining of tdTomato at different growth stages of hair follicles following depilation in *Krt24-Cre^ERT2^*;*Rosa26^LSL-tdTomato^* mice after TAM treatment. *n* = 3 mice/time point. (**E**) *Krt24-Cre^ERT2^*;*Rosa26^LSL-tdTomato^* mice had their hair follicles induced to enter the growth phase and were then treated with TAM for 5 consecutive days, and no tdTomato positive signal was detected in hair follicles in the growth phase. *n* = 3 mice. Scale bar, 100 μm. TAM, tamoxifen; ORS: outer root sheath.

To study the spatial pattern of *Krt24^+^* cells during HF development, we crossed *Krt24-Cre^ERT2^* with the Cre-dependent reporter mouse line *Rosa26^LSL-tdTomato^* (*Rosa26^loxP-stop-loxP-tdTomato^*). In *Krt24-Cre^ERT2^*;*Rosa26^LSL-tdTomato^* mice (Figure 2A), Cre is fused with the mutant ligand-binding domain of ER (ERT2). This mutation cannot bind to endogenous estrogen but can bind to tamoxifen (TAM), a selective estrogen receptor modulator. In the absence of TAM, Cre^ERT2^ protein is retained in the cytoplasm. However, upon TAM treatment, TAM binds to ERT2, enabling Cre to translocate into the nucleus. Cre recombinase then deletes the STOP sequence flanked by two loxP sites, resulting in the permanent expression of tdTomato.

To assess the activity of Cre and check for any potential leakiness, *Krt24-Cre^ERT2^*;*Rosa26^LSL-tdTomat^* mice were administered TAM via intraperitoneal injection at a dose of 80 mg/kg for five consecutive days, while the control group received 0.9% NaCl. Subsequently, the dorsal skins were collected for immunofluorescence staining. Without TAM induction, tdTomato signals were absent in hair follicles (Figure 2B). However, a significantly positive tdTomato signal was detected in the outer cells of bulge after TAM treatment (Figure 2C). These results demonstrate that Cre activation occurs strictly in the presence of TAM and that Cre-mediated recombination is restricted to cells residing in the bulge region of mouse dorsal skins.

After the hair follicle enters the anagen phase, the stem cells in the bulge region are activated and extensively proliferate, moving downward to form the outer root sheath (ORS) structure [31]. To verify whether the tdTomato^+^ cells located in the bulge region, marked in our experiment, would undergo the same changes, we treated 6- to 8-week-old *Krt24-Cre^ERT2^*;*Rosa26^LSL-tdTomat^* mice (the dorsal skin HFs of which are usually in the telogen phase, when *Krt24* is expressed in OB HFSCs) with TAM for five consecutive days, followed by wax depilation to induce the entrance of HFs into the anagen phase. Then, we collected dorsal skins at 0, 7, 14, 21, and 28 days postdepilation, prepared paraffin sections, and performed immunofluorescence staining assays (Figure 2A). Similarly, we observed tdTomato^+^ cells mainly located in the telogen bulge of HF, which then expanded to the ORS as the HFs entered the anagen phase (Figure 2D). Importantly, from the anagen to next telogen phase, tdTomato expression was also maintained in the offspring of *Krt24^+^* HFSCs in the bulge region, which may ensure the stem cell availability for the next HF cycle (Figure 2D).

To further verify that tdTomato^+^ cells entering the anagen phase originate from *Krt24^+^* bulge HFSCs in the telogen phase, we depilated a middle area of the dorsal skin in *Krt24-Cre^ERT2^*;*Rosa26^LSL-tdTomat^* mice to induce their hair follicles into the growth cycle. Seven days later, we administered TAM to the mice via intraperitoneal injection for five consecutive days. At 14 days postdepilation, we collected the dorsal skins of the mice for immunofluorescence staining. However, we did not observe a tdTomato^+^ signal in anagen-phase HFs (Figure 2E). In summary, using *Krt24-Cre^ERT2^*;*Rosa26^LSL-tdTomat^* mice, we specifically labeled *Krt24^+^* cells located in the outer bulge region (Figure 2C,D). These cells exhibit characteristics of OB HFSCs because they can be rapidly activated and develop into the ORS structure once the hair follicle enters the anagen phase and can persist into the next telogen phase (Figure 2D). Additionally, *Krt24* expression is low after entering the anagen phase (Figure 2E), which is consistent with the conclusion from a previous study [16].

### 2.3. TdTomato-Labeled Krt24^+^ Cells Belong to Outer Bulge Hair Follicle Stem Cells

**Figure 3:**
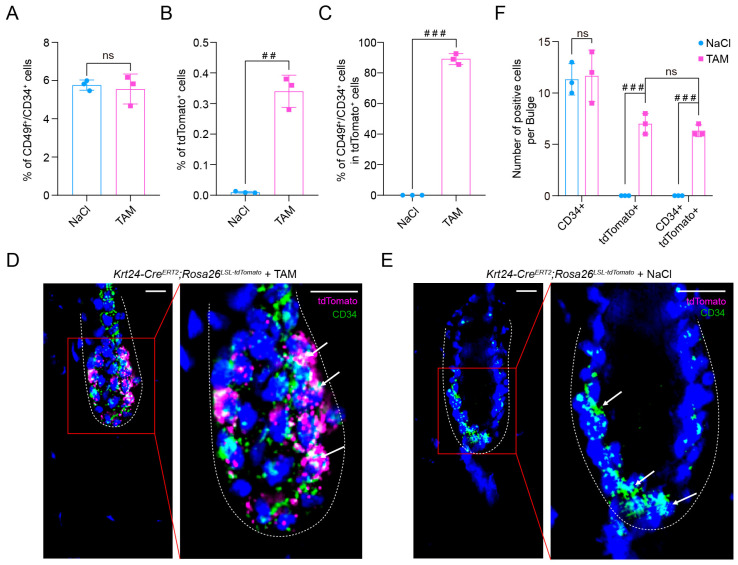
TdTomato-Labeled *Krt24^+^* Cells Belong to Outer Bulge Hair Follicle Stem Cells. (**A**–**C**): Flow cytometry analysis of dorsal skin from TAM- or 09% NaCl-treated *Krt24-Cre^ERT2^;Rosa26^LSL-tdTomato^* mice showing: (**A**), Percentage of CD49f^+^/CD34^+^ cells (Unpaired Student’s *t*-test). *n* = 3 mice/group. (**B**), Percentage of tdTomato^+^ cells (Welch’s *t*-test, ^##^ *p* < 0.01). *n* = 3 mice/group. (**C**), Percentage of CD49f^+^/CD34^+^ cells within tdTomato^+^ cells (Mann Whitney test, ^###^ *p* < 0.05). *n* = 3 mice/group. (**D**) After TAM treatment for 5 consecutive days, immunofluorescence costaining of tdTomato and the bulge HFSC marker CD34 was performed in telogen-phase hair follicles of *Krt24-Cre^ERT2^;Rosa26^LSL-tdTomato^* mice. *n* = 3 mice. (**E**) After 0.9% NaCl treatment for 5 consecutive days, immunofluorescence costaining of tdTomato and the bulge HFSC marker CD34 was performed in telogen-phase hair follicles of *Krt24-Cre^ERT2^;Rosa26^LSL-tdTomato^* mice. *n* = 3 mice. (**F**) After TAM or 0.9% NaCl treatment, immunofluorescence costaining of tdTomato and the bulge HFSC marker CD34 was performed in telogen-phase hair follicles of *Krt24-Cre^ERT2^;Rosa26^LSL-tdTomato^* mice. The number of positive cells was quantitatively analyzed by ImageJ. For each sample, three randomly selected fields were analyzed, with positive cells quantified in 3 hair follicles per field (3 fields × 3 follicles). (Mann Whitney test, ^###^ *p* < 0.001). *n* = 3 mice/group. Scale bars, 10 μm. TAM: tamoxifen.

To examine whether *Krt24* can accurately mark telogen OB HFSCs, we further investigated their biological characteristics. Given the high heterogeneity of gene expression of OB HFSCs during telogen and anagen phases, the standard labeling markers are CD34 and CD49f, effectively demonstrated in multiple flow cytometry or immunofluorescence experiments [32]. To further characterize the tdTomato^+^ cells, we used this “gold standard” for identifying OB HFSCs. We intraperitoneally administered TAM or 0.9% NaCl to *Krt24-Cre^ERT2^*;*Rosa26^LSL-tdTomat^* mice for five consecutive days, then collected dorsal skins from mice with hair follicles in the telogen phase and performed flow cytometry analyses and immunofluorescence staining assays. In the dorsal skin of *Krt24-Cre^ERT2^*;*Rosa26^LSL-tdTomat^* mice treated with TAM or 0.9% NaCl, CD34 in hair follicles showed no difference (Figure 3A). But in the 0.9% NaCl-treated group, tdTomato^+^ cells were barely detectable (Figure 3B,E). Importantly, results revealed that over 90% of tdTomato^+^ cells were double-positive for CD34 and CD49f. (Figure 3C and Supplementary Figure S2). Consistently, immunofluorescence staining showed that all tdTomato^+^ cells within the outer bulge compartment exhibited colocalization with CD34 (Figure 3D,F). Taken together, our findings demonstrate that tdTomato-labeled *Krt24^+^* cells are predominantly localized within the OB HFSCs compartment. These data establish the *Krt24-Cre^ERT2^*;*Rosa26^LSL-tdTomat^* mouse line as a reliable genetic tool for in vivo tracing of OB HFSCs dynamics during hair follicle morphogenesis and cutaneous wound repair processes.

### 2.4. Generation of the Krt24-Cre^ERT2^;Rosa26^LSL-DTR^ Mouse Model

**Figure 4:**
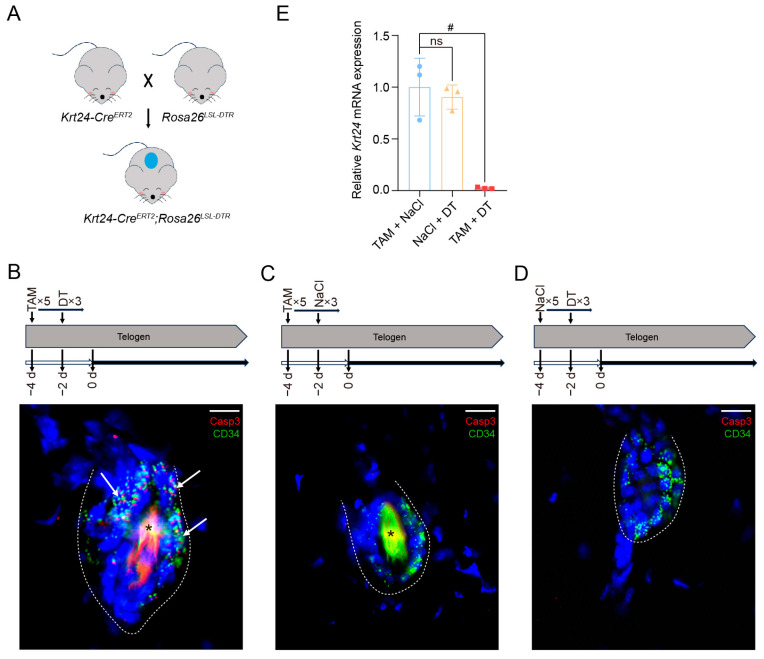
Generation of the *Krt24-Cre^ERT2^*;*Rosa26^LSL-DTR^* mouse model. (**A**) Generation of *Krt24-Cre^ERT2^*;*Rosa26^LSL-DTR^* mice by crossing *Krt24-Cre^ERT2^* mouse line with the Cre-inducible ablation line *Rosa26^LSL-DTR.^.* (**B**) Mice were treated with TAM for 2 consecutive days, followed by TAM and DT for 3 consecutive days. Immunofluorescence costaining of Casp3 and CD34 (a bulge hair follicle stem cell marker) was performed on telogen-phase dorsal skin samples from *Krt24-Cre^ERT2^*;*Rosa26^LSL-DTR^* mice. *n* = 3 mice. (**C**) Mice were treated with TAM for 2 consecutive days, followed by TAM and 0.9% NaCl for 3 consecutive days. Immunofluorescence costaining of Casp3 and CD34 was performed on telogen-phase dorsal skin samples from *Krt24-Cre^ERT2^*;*Rosa26^LSL-DTR^* mice. *n* = 3 mice. (**D**) Mice were treated with 0.9% NaCl for 2 consecutive days, followed by 0.9% NaCl and DT for 3 consecutive days. Immunofluorescence costaining of Casp3 and CD34 was performed on telogen-phase dorsal skin samples from *Krt24-Cre^ERT2^*;*Rosa26^LSL-DTR^* mice. *n* = 3 mice. (**E**) *Krt24* transcript levels (normalized to Actb) in telogen-phase dorsal skin of *Krt24-Cre^ERT2^*;*Rosa26^LSL-DTR^* mice treated with TAM, DT, or 0.9% NaCl, analyzed by qRT-PCR 24 h post-final DT treatment (Welch’s *t*-test, ^#^ *p* < 0.05). Asterisks mark autofluorescence from hair shafts. *n* = 3 mice/group. Scale bar, 10 μm. TAM: tamoxifen; DT: diphtheria toxin.

To investigate the biological functions of *Krt24^+^* cells, we generated *Krt24-Cre^ERT2^*;*Rosa26^LSL-DTR^* mice through crossing *Krt24-Cre^ERT2^* mouse line with the Cre-inducible ablation line *Rosa26^LSL-DTR^* (Figure 4A). In this dual-recombinase system, TAM-induced Cre activation mediates permanent excision of the loxP-flanked STOP cassette, enabling the constitutive expression of diphtheria toxin receptor (DTR) in target cells. Subsequent administration of diphtheria toxin (DT) triggers caspase-dependent apoptosis specifically in cells expressing DTR.

To induce the deletion of *Krt24*^+^cells, *Krt24-Cre^ERT2^*;*Rosa26^LSL-DTR^* mice were intraperitoneally administered TAM for five consecutive days and DT for three successive days prior to experimentation. Vehicle control groups received equivalent volumes of 0.9% NaCl. Equally, in order to exclude potential confounding effects of individual treatments, we also treated *Krt24-Cre^ERT2^*;*Rosa26^LSL-DTR^* mice with 0.9% NaCl and DT. Dorsal skins were subsequently collected for comparative analyses using immunofluorescence staining and quantitative real-time polymerase chain reaction (qRT-PCR) assays. After TAM and DT administration to *Krt24-Cre^ERT2^*;*Rosa26^LSL-DTR^* mice, a strong cleaved Caspase3 signal was observed in the OB region, marked by the bulge marker CD34 (Figure 4B), indicating a marked induction of *Krt24*^+^ cell apoptosis. In contrast, no apoptotic cells were detectable in *Krt24-Cre^ERT2^*;*Rosa26^LSL-DTR^* mice without DT or TAM treatment (Figure 4C,D). qRT-PCR analysis further confirmed that *Krt24* mRNA was significantly downregulated in the back skin of *Krt24-Cre^ERT2^*;*Rosa26^LSL-DTR^* mice treated with TAM and DT (Figure 4E). However, there was no significant change in *Krt24* expression in the dorsal skin of *Krt24-Cre^ERT2^*;*Rosa26^LSL-DTR^* mice treated with TAM or DT only. (Figure 4E).

Collectively, the *Krt24-Cre^ERT2^*;*Rosa26^LSL-DTR^* model enables targeted and efficient ablation of *Krt24^+^* stem cells within the hair follicle bulge niche. This system provides a spatiotemporally controlled platform to study the functional contributions of *Krt24^+^* cells during hair follicle morphogenesis and cutaneous wound repair processes.

### 2.5. Ablation of Krt24^+^ Cells Significantly Delays the Telogen-to-Anagen Transition in Hair Follicle Cycling

**Figure 5:**
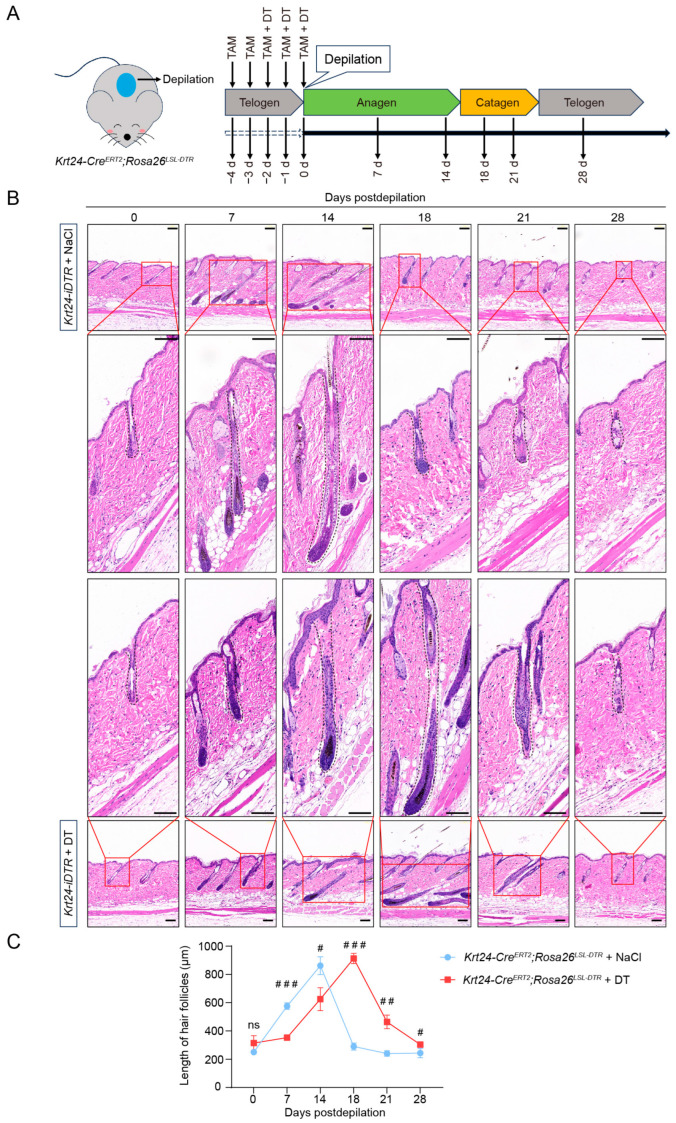
Ablation of *Krt24^+^* cells significantly delays the telogen-to-anagen transition in hair follicle cycling. (**A**) *Krt24-Cre^ERT2^*;*Rosa26^LSL-DTR^* mice were administered TAM for 2 consecutive days, followed by combined TAM and DT treatment for 3 consecutive days. The control group received an equivalent volume of TAM and 0.9% NaCl. Depilation was then performed to synchronize hair follicles into the anagen phase. Dorsal skin samples were collected at 0, 7, 14, 21, and 28 days postdepilation for H&E staining. (**B**) H&E staining of the dorsal skins from *Krt24-Cre^ERT2^*;*Rosa26^LSL-DTR^* mice at different developmental stages of hair follicles. *n* = 3 mice/group/time point. (**C**) Statistical analyses of hair follicle length in the dorsal skins of *Krt24-Cre^ERT2^*;*Rosa26^LSL-DTR^* mice at different developmental stages of hair follicles. For each sample, 3 random visual fields were analyzed, with 3 hair follicle lengths measured per field (3 fields × 3 follicles). (Unpaired Student’s *t*-test, ^#^ *p* < 0.05, ^##^ *p* < 0.01, ^###^ *p* < 0.001.) *n* = 3 mice/group/time point. Scale bars, 100 μm. TAM, tamoxifen; DT, diphtheria toxin; H&E: hematoxylin and eosin.

Next, we tested whether ablation of *Krt24^+^* stem cells has a functional impact on hair follicle homeostasis. We treated 6- to 8-week-old *Krt24-Cre^ERT2^*;*Rosa26^LSL-DTR^* mice with TAM for two consecutive days, followed by TAM and DT treatment for three consecutive days (Figure 5A). And the control group received TAM and 0.9% NaCl injections. Then, the hair shafts were mechanically depilated to induce the hair follicles to enter the growth cycle, and the skins were collected for hematoxylin and eosin (H&E) staining (Figure 5A).

Seven days postdepilation, the hair follicles of *Krt24-Cre^ERT2^*;*Rosa26^LSL-DTR^* mice treated with 0.9% NaCl extended downward in growth, indicating that the hair follicles had entered the anagen phase. At 14 days postdepilation, the newly formed hair shafts reached their maximum length and covered the entire skin area (Figure 5B,C). Subsequently, the hair follicles began to rapidly shorten until day 21, at which point the hair follicle length returned to a level equal to that in the telogen phase (Figure 5B,C). This stage corresponded to the catagen phase in hair follicle development. From day 21 to day 28, there was no significant change in hair follicle length, indicating the reentry of hair follicles into the telogen phase (Figure 5B,C). The progression of the hair follicle growth cycle in *Krt24-Cre^ERT2^*;*Rosa26^LSL-DTR^* mice was consistent with previous reports [33], and this also indicates that the normal development of hair follicles of the hybrid mouse model was not adversely affected without DT treatment.

Conversely, *Krt24-Cre^ERT2^*;*Rosa26^LSL-DTR^* mice treated with DT exhibited a different pattern of hair follicle regeneration. At 7 days postdepilation, these mice did not show significant elongation of hair follicles (Figure 5B,C). Hair follicles in the anagen phase became evident on day 14, with the newly formed hair shafts reaching maximum length by day 18 (Figure 5B,C). The transition to the catagen phase was further delayed, with hair follicles in this stage first observed on day 21 postdepilation (Figure 5B,C). Ultimately, the hair follicle length in DT-treated *Krt24-Cre^ERT2^*;*Rosa26^LSL-DTR^* mice returned to a level comparable to that in the telogen phase on day 28 (Figure 5B,C). Compared with the control group, although there were no significant changes in the maximum length achieved during the anagen phase and the hair follicle structure in *Krt24^+^* cell-depleted mice, the hair follicles lacking *Krt24^+^* cells failed to promptly initiate the anagen phase after depilation, leading to a delay in the hair follicle growth cycling.

### 2.6. Krt24^+^ OB HFSC Response to Ionizing-Radiation-Induced Skin Damage

**Figure 6:**
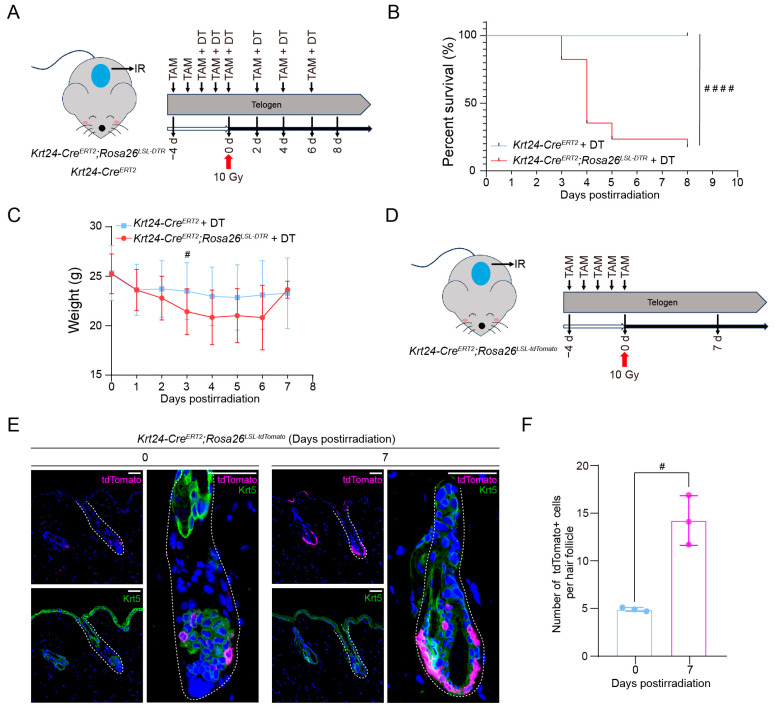
*Krt24^+^* OB HFSC response to ionizing-radiation-induced skin damage. (**A**) Experimental flow diagram for assessing the effects of *Krt24^+^* cell depletion on the response of mice to ionizing radiation using *Krt24-Cre^ERT2^*;*Rosa26^LSL-DTR^* mice. Both *Krt24-Cre^ERT2^*;*Rosa26^LSL-DTR^* and *Krt24-Cre^ERT2^* mice were administered TAM for 2 consecutive days, followed by combined TAM and DT treatment for 3 consecutive days to eliminate *Krt24^+^* cells. The dorsal skin was then locally irradiated with 10 Gy γ-ray from ^60^Co. Subsequently, TAM and DT were administered every two days, and the mice’s body weight and survival status were recorded daily. (**B**) The changes in survival time for irradiated mice (log-rank test, ^####^ *p* < 0.0001). *n* = 18 mice/group. (**C**) The changes in body weight for irradiated mice (Mann–Whitney U test, ^#^ *p* < 0.05). *n* = 18 mice/group. (**D**) Experimental flow diagram for tracing the dynamics of *Krt24^+^* cells using *Krt24-Cre^ERT2^;Rosa26^LSL-tdTomato^* mice. The mice were administered TAM for 5 consecutive days. The dorsal skin was then locally irradiated with a 10 Gy γ-ray from ^60^Co. Dorsal skin samples were collected at 0 and 7 days postradiation for immunofluorescence staining assays. (**E**) Without depilation, the dorsal skins of *Krt24-Cre^ERT2^*;*Rosa26^LSL-tdTomato^* mice were subjected to 10 Gy of ionizing radiation, followed by immunofluorescence costaining for tdTomato and the stem cell marker *Krt5*. *n* = 3 mice/time point. (**F**) Quantification of tdTomato^+^ cells in hair follicles was performed at 0 and 7 days postirradiation. For each sample, three randomly selected fields were analyzed, with tdTomato⁺ cells quantified in 3 hair follicles per field. *n* = 3 mice/time point. (Welch’s *t*-test, ^#^ *p* < 0.05). Scale bars, 50 μm. TAM, tamoxifen; DT, diphtheria toxin.

Given the pivotal role of *Krt24^+^* OB HFSCs in HF development, we further investigated the function in hair follicle homeostasis after strong damage (e.g., ionizing radiation). 6- to 8-week-old *Krt24-Cre^ERT2^*;*Rosa26^LSL-DTR^* mice received intraperitoneal TAM injections daily for two consecutive days. Subsequently, TAM and DT were coadministered for three consecutive days to eliminate *Krt24^+^* cells. After that, the middle area of the dorsal skin was exposed to 10 Gy of ionizing radiation from a ^60^Co radiation source. Following irradiation, TAM and DT were administered every two days to continuously deplete *Krt24^+^* cells (Figure 6A). To rule out the intergroup impact caused by the continuous injection of TAM and DT, the control *Krt24-Cre^ERT2^* mice, lacking DTR expression, underwent identical TAM/DT administration and irradiation protocols. The body weight and survival status of the mice were then monitored daily. The results showed that mice with continuous depletion of *Krt24^+^* HFSCs exhibited a more significant decrease in body weight after ionizing radiation exposure and experienced substantial mortality within 3 to 5 days postirradiation (dpi) (Figure 6B,C).

To further explore the changes that occur in *Krt24^+^* OB HFSCs after irradiation, we used *Krt24-Cre^ERT2^*;*Rosa26^LSL-tdTomat^* mice to tracing the dynamics of *Krt24^+^* OB HFSCs. *Krt24-Cre^ERT2^*;*Rosa26^LSL-tdTomat^* mice were administered with TAM for five consecutive days. Subsequently, the middle area of their dorsal skin was exposed to 10 Gy of ionizing radiation from a ^60^Co radiation source (Figure 6D). Dorsal skin specimens from irradiated areas were harvested at 0 and 7 days postirradiation for multiplex immunofluorescence analyses. Quantitative analyses demonstrated a marked increase in tdTomato^+^ cell populations within irradiated hair follicles at 7 days postexposure. Spatial mapping revealed these fluorescently tagged cells localized preferentially to the inferior and central subdomains of the outer bulge niche (Figure 6E,F). Additionally, tdTomato colocalized with the keratinocyte marker *Krt5* (Figure 6E), indicating that tdTomato^+^ cells possess the ability to renew and proliferate although they didn’t enter the anagen phase. Taken together, these findings indicate that *Krt24^+^* OB HFSCs play a pivotal role in tissue repair and stress response.

## 3. Discussion

KRT24, encoded by the *Krt24* gene, is a member of the type I keratin family [9]. Previous studies have demonstrated its stage-specific expression pattern during hair follicle morphogenesis, with showing significantly higher expression in telogen-phase OB HFSCs than in those in the anagen/catagen phases [16,17,22]. While the functional significance of *Krt24*^+^ cells in follicular biology remains poorly understood, emerging evidence suggests their potential involvement in stem cell regulation. Notably, epidermal wounding experiments have revealed that superficial injuries confined to the epidermis can activate *Krt24^+^* bulge HFSCs, inducing their rejuvenation [34]. To elucidate the functional role of *Krt24^+^* OB HFSCs within the dynamic hair follicle niche, we generated a tamoxifen-inducible *Krt24-Cre^ERT2^* mouse line by using CRISPR/Cas9 to knock in *Cre^ERT2^* into exon 8 of the *Krt24* gene (Figure 1A). This genetic strategy successfully achieved spatiotemporal specificity in labeling OB HFSCs, as demonstrated by our characterization studies. The established model provides three key advantages: (1) precise temporal control over genetic manipulation through tamoxifen induction; (2) spatial restriction to the outer bulge compartment; (3) functional preservation of endogenous *Krt24* expression.

Multiple transgenic mouse lines have been developed to label HFSCs through keratin family genes. These models help elucidate the roles of HFSCs in skin homeostasis and disease. Wang et al. [25] employed *Krt5-Cre^ERT2^* mediated conditional knockout to ablate *OSMRβ* or *STATa/b* in keratin 5-expressing epidermal and HFSCs. This genetic perturbation induced premature telogen-to-anagen transition in HFSCs, establishing the *OSMRβ-STAT5* signaling axis as a critical regulator of stem cell quiescence maintenance. Lisse et al. [35] employed an intersectional genetics approach by crossing *Krt14-Cre^ERT2^* mice (*Krt14* promoter-driven *Cre^ERT2^* mice) with *Ret^flox/flox^* mice to generate *Krt14-Cre^ERT2^;Ret^flox/flox^* mouse line. After treatment with TAM, the activity of Cre recombinase was induced. This led to the specific knockout of the *Ret* gene in basal keratinocytes and the outer epidermal layer cells of hair follicles. While both models achieve spatiotemporal control of Cre activity through TAM induction, the *Krt5/Krt14-Cre* lineages exhibit broad tropism encompassing basal epidermal progenitors and heterogeneous HFSC populations. This pancompartmental targeting complicates precise interrogation of functionally distinct cellular subpopulations within the hair follicle niche. Keratin 15 (KRT15), encoded by the *Krt15* gene, is predominantly localized to the bulge and HG compartments during telogen [3]. The mifepristone (RU486)–inducible *Krt15-Cre^PR^* system has enabled targeted genetic manipulation in bulge-resident HFSCs, providing critical insights into their roles in maintaining follicular homeostasis and orchestrating injury repair processes [36,37,38]. Complementing this approach, *Lgr5-Cre^ERT2^* mice label cycling stem cell populations through tamoxifen induction, targeting both telogen bulge cells and anagen-phase ORS progenitors [5,36,39]. This model has proven particularly valuable for tracking transient activation states during hair cycle progression.

Although *Krt5*, *Krt14*, and *Krt15* remain the most widely utilized markers for HFSCs, their application faces two principal limitations. First, these keratins exhibit variable specificity across HFSC subpopulations. Second, their expression patterns show temporal heterogeneity during distinct phases of the hair follicle cycle. Specifically, *Krt5* and *Krt14* demonstrate pan-HFSC expression throughout multiple developmental stages, particularly during anagen and catagen [17,22,40,41,42]. In contrast, *Krt15* expression localizes to HFSCs within the bulge and HG regions, showing predominant activity during anagen and telogen [36,43,44]. Notably, *Sox9* and *Lgr5* are not uniquely expressed by bulge cells but rather extend to the HG and lower outer root sheath of cycling follicles [40]. This overlapping expression profile limits precise spatial mapping of functionally distinct HFSC subsets. Recent evidence has identified *Krt24* as a superior marker, showing restricted expression in OB HFSCs during telogen. This spatially constrained expression pattern positions *Krt24* as an optimal tool for investigating OB HFSC contributions to follicular homeostasis. While these models differ in spatiotemporal resolution and targeting specificity, their collective application has significantly advanced our understanding of HFSC heterogeneity and regenerative plasticity. The complementary strengths of these systems have been instrumental in mapping stem cell behaviors across distinct physiological states, from homeostasis to injury response.

The *Krt24-Cre^ERT2^* mouse line generated in this study has high spatiotemporal targeting specificity. It must be acknowledged that the molecular mechanisms underlying the observed cyclical expression dynamics of *Krt24* remain enigmatic. However, such phenomena are not without precedent in cutaneous stem cell biology. Keratins, while widely employed as lineage-specific markers [23,27,28], are rarely implicated in certain signal transduction cascades. Notably, the regulatory basis for their compartmentalized expression patterns remains largely undefined, yet these markers retain broad utility in stem cell characterization [42,43,44]. Analogously, our work identifies *Krt24* as a keratin-based marker selectively expressed in quiescent OB HFSCs, a designation corroborated by multiple independent studies [18,19,20,21].

Current paradigms posit that OB HFSC quiescence is primarily regulated by BMP signaling [45], with Wnt activation serving as a prerequisite for their proliferative entry [41]. While ancillary pathways (e.g., FGF, Shh) have been implicated in OB HFSC activation [1], a comprehensive molecular atlas delineating their transitions across anagen activation and catagen regression remains elusive. Our newly established *Krt24-Cre^ERT2^* mouse line allows for precise targeting of *Krt24^+^* cells and their progeny during hair follicle dynamic remodeling. This tool helps clarify the molecular mechanisms underlying the transition of hair follicle stem cells from the telogen to anagen phase and explores *Krt24*’s role in this process. Additionally, this model can be crossed with reporter mice (such as *Rosa26^LSL-tdTomato^*, *Rosa26^LSL-DTR^*) to perform lineage-tracing of quiescent bulge HFSCs in hair follicle regeneration and wound repair and further reveal their differentiation potential [34,46,47,48]. Furthermore, this model has the application potential to study how abnormal function of bulge HFSCs leads to hair follicle atrophy or regenerative disorders [35,49], and to study the role of bulge HFSCs in skin tumors [50,51].

Adverse skin reactions are one of the side effects of radiotherapy in clinical practice [52]. Hair follicles are highly sensitive to ionizing radiation, and the radiosensitivity varies among different compartments of the hair follicle [53]. Quiescent bulge HFSCs exhibit the lowest radiation sensitivity [54]. Moreover, these cells express higher levels of the anti-apoptotic gene *Bcl2* and show dampened p53 activation [55]. Our lineage tracing studies revealed that *Krt24^+^* cells specifically reside within the bulge compartment of hair follicles (Figure 2C), exhibiting characteristic molecular signatures of outer bulge HFSCs (CD34^+^/CD49f^+^) (Figure 3C and Supplementary Figure S2). Intriguingly, these cells displayed dynamic plasticity during follicular cycling (Figure 2D), suggesting their potential role as a responsive reservoir upon stem cell niche perturbation. To test this hypothesis, we subjected mice to ionizing-radiation-induced cutaneous injury. A significant proliferative response was observed in *Krt24^+^* cells at 7 days postirradiation (Figure 6E,F). Previous studies have also demonstrated that hair follicle epithelial stem cells located in the bulge region exhibit significant proliferation five days after radiation exposure [54]. In our experiments, mice with ablation of *Krt24^+^* cells exhibited significantly reduced survival periods and body weight following ionizing radiation exposure (Figure 6B,C), which further suggests that this cell population plays a critical role in ionizing radiation-induced hair follicle damage.

Notably, compared with existing Cre mouse lines targeting HFSCs, the *Krt24-Cre^ERT2^* mouse line demonstrates enhanced precision in labeling the OB HFSCs. However, considering the low proportion of OB HFSCs among all skin cells [56], this necessitates methodological optimization to enhance labeling efficiency, such as optimizing the dose and frequency of TAM administration.

Taken together, we established a *Krt24-Cre^ERT2^* mouse line enabling the specific labeling of telogen OB HFSCs in skins. This novel Cre line will facilitate studies on the fate of OB HFSCs during hair follicle cycling and in response to injury.

## 4. Materials and Methods

### 4.1. Mice

All animal experiments were approved by and conducted under the supervision of the Animal Care and Use Committee of the Beijing Institute of Radiation Medicine (approval number: IACUC-DWZX-2022-792). The mice were maintained in specific pathogen-free (SPF) facilities with a 12-h light/dark cycle and had free access to food and water. For this study, male mice aged 6–8 weeks were utilized, including *Rosa26^LSL-tdTomato^* (Cat.# C001181), *Rosa26^LSL-DTR^* (Cat.# C001189), and *Krt24-Cre^ERT2^* strains, all of which were obtained from Cyagen Biosciences Inc. (Suzhou, China). All experimental mice were maintained on a C57BL/6J genetic background to ensure genetic consistency across the study groups. Surgical anesthesia was maintained via a precision isoflurane vaporizer (2% concentration in 4 L/min fresh gas flow at 0.41 mL/min delivery rate) with continuous respiratory monitoring. Humane endpoints (skin ulceration) triggered immediate CO_2_ euthanasia.

### 4.2. Generation and Genotyping of Krt24-Cre^ERT2^ Knock-in Mice

The *Krt24*-*Cre^ERT2^* knock-in mouse line was generated through CRISPR/Cas9-mediated genome editing. Specifically, the single guide RNA (gRNA) targeting the mouse *Krt24* gene (5′-CACCAGACTGACCATTGTGCCGG-3′), along with a donor vector containing the “IRES-CreERT2-WPRE-rBG polyA” cassette and Cas9 mRNA, were microinjected into mouse zygotes. The cap-independent translation driven by internal ribosomal entry sites (IRESs) can serve as an alternative mechanism for protein production. Founder (F0) animals were identified by PCR and subsequent sequence analysis, and the positive founders were subsequently bred with wild-type C57BL/6J mice to establish the F1 generation. The following primer pairs were used to identify the target allele in the constructed *Krt24*-*Cre^ERT2^* mice: F1 (5′-CCAATGCCCTGGCTCACAAATAC-3′), R1 (5′-GTTGGGACCCACAGCAAGAAAAC-3′), F2 (5′-CAGATTGAGGGTCTGACTGAGGAG-3′), R2 (5′-CAAAAGACGGCAATATGGTGGAAAA-3′), F3 (5′-AAGACAAGGGTGACAAAGACCATC-3′), R3 (5′-AAATGAGCTGTCAATCATGACTCC-3′), F4 (5′-AGACAAGGGTGACAAAGACCATCATA-3′), R4 (5′-GACCTTGCATTCCTTTGGCGA-3′). The PCR product of primers F1/R1 includes the downstream region of the 3′arm and part of the inserted donor DNA. The PCR product of primers F2/R2 includes the upstream region of the 5′arm and part of the inserted donor DNA. Therefore, primers F1/R1 and F2/R2 detect the presence of inserted donor DNA in heterozygous and homozygous mice. To optimize genotyping of the *Krt24-Cre^ERT2^* mice, primers F4/R4 were designed to amplify the inserted DNA, and primers F3/R3 were designed to amplify the wild-type allele.

### 4.3. Genomic DNA Extraction and Genotyping Analysis

Genomic DNA was isolated from 2-week-old mouse tail biopsies (0.3–0.5cm) using the Tail Genomic DNA Extraction Kit (CW2094S, CWBio, Taizhou, Jiangsu, China). The PCR amplification was performed using KOD polymerase (KOD-401, TOYOBO, Kita-ku, Osaka-shi, Japan), followed by agarose gel electrophoresis for DNA product size verification. The following primer pairs were used to genotype the *Rosa26^LSL-tdTomato^* mice: F1 (5′-GGCATTAAAGCAGCGTATCC-3′), R2 (5′-CTGTTCCTGTACGGCATGG-3′), F3 (5′-AAGGGAGCTGCAGTGGAGTA-3′), R4 (5′-CCGAAAATCTGTGGGAAGTC-3′), with F1/R2 amplifying the target allele and F3/R4 amplifying the wild-type allele. The following primer pairs were used to genotype the *Rosa26^LSL-DTR^* mice: F1 (5′-CCTATGACCATACAACTATCCTGGC-3′), R1 (5′-GGGTGAGCATGTCTTTAATCTACC-3′), F2 (5′-CACTTGCTCTCCCAAAGTCGCTC-3′), R1 (5′-GGGTGAGCATGTCTTTAATCTACC-3′), with F1/R1 amplifying the target allele and F2/R1 amplifying the wild-type allele. All PCR reactions were performed under standardized conditions: initial denaturation at 94 °C for 5 min, followed by 35 cycles of denaturation (94 °C, 30 s), annealing (60 °C, 30 s), and extension (72 °C, 1 min), with a final extension at 72 °C for 10 min.

### 4.4. Southern Blot Assays

Southern blot analysis was performed to validate successful gene targeting in five F1 mice. Genomic DNA was digested with restriction endonucleases ScaI (R3156V, New England Biolabs, Ipswich, MA, USA) and BstEII (R3162V, New England Biolabs, Ipswich, MA, USA) followed by electrophoresis in 0.8% agarose gel. The resolved DNA fragments were denatured in situ using alkaline solution (0.5 M NaOH/1.5 M NaCl), and the single-stranded DNA fragments were transferred to nylon membranes (RPN1510B, Cytiva, Marlborough, MA, USA). DNA fixation was achieved by ultraviolet crosslinking (03-II, SCIENTZ, Ningbo, Zhejiang, China). Prehybridization was conducted in UltraHyb-Oligo buffer (AM8663, Invitrogen, Carlsbad, CA, USA) at 65 °C for 1 h. Denatured probes (50 ng/mL) were hybridized for 16 h and then washed. Subsequent detection was performed using Anti-Digoxigenin-AP, Fab fragments from sheep (11093274910, Roche, South San Francisco, CA, USA) for chemiluminescence detection. Signals were captured using an Imaging System (5200, Tanon, Shanghai, Shanghai, China) with optimized exposure times.

DIG-conjugated probes were amplified using the PCR DIG Probe Synthesis Kit (11636090910, Roche, South San Francisco, CA, USA) according to the manufacturer’s thermal profile: 94 °C for 2 min; 35 cycles of 94 °C 30 s, 60 °C 30 s, 72 °C 20 s; final extension at 72 °C for 7 min. Amplification specificity was confirmed by agarose gel electrophoresis (1.5%) prior to hybridization. KI probe forward primer: 5′-TGCCCTGGCTCACAAATACCACT-3′, KI probe reverse primer: 5′-TAGCCAACCTTTGTTCATGGCAGC-3′.

### 4.5. Tamoxifen Induction and Diphtheria Toxin-Induced Cell Ablation

Mice were induced with tamoxifen (TAM; S1238, Selleck, Houston, TX, USA) to activate Cre activity, with injections continuing for 5 days up to the depilation or ionizing radiation (IR). *Krt24-Cre^ERT2^*;*Rosa26^LSL-DTR^* mice received three consecutive daily diphtheria toxin (DT; #150, List Labs, Campbell, CA, USA) injections prior to depilation/irradiation to deplete target cells. In particular, *Krt24-Cre^ERT2^*;*Rosa26^LSL-DTR^* mice were injected with TAM and DT every two days after IR until the end of the experiment. For mice requiring ionizing irradiation, the animals were positioned with their dorsal side facing upward on a plate rack. Subsequently, regions not intended for irradiation were shielded using lead blocks to ensure precise targeting. Then, the mice were exposed to ionizing radiation from ^60^Co source and received a total dose of 10 Gy (1.5 cm × 1.5 cm dorsal field). The 10 Gy dose was chosen for its proven ability to induce significant hair follicle damage [57]. TAM was dissolved in corn oil at a final concentration of 20 mg/mL. Each mouse was administered at a dose of 80 mg/kg body weight via intraperitoneal injection [25,37,48]. DT was dissolved in phosphate-buffered saline (PBS) at a concentration of 100 μg/mL to prepare a 10 × stock solution. Before use, the stock solution was diluted to 1 × with PBS. Each mouse was administered at a dose of 1 μg/100 μL via intraperitoneal injection.

### 4.6. Tissue Collection

After TAM and DT administration, the hair on the designated area of the dorsal skin was removed with wax. This was done to induce the hair follicle growth cycle. Mouse dorsal skin was collected at the specified time points. Appropriate-sized whole skin tissues were collected under anesthesia with 1% pentobarbital sodium, spread out, and fixed overnight at 4 °C in 4% paraformaldehyde (PFA).

### 4.7. Immunofluorescence Staining

Tissue samples fixed with 4% PFA were dehydrated and clarified before being paraffin-embedded, and 10 μm sections were cut. After dewaxing and rehydration, antigen retrieval was performed. The tissue sections were blocked with 10% goat serum for 20 min at room temperature, then incubated with the primary antibody overnight at 4 °C. The sections were then washed three times with PBS and incubated with the secondary antibody for 2 h at room temperature. Then, the sections were incubated with DAPI for 10 min at room temperature to stain the cell nuclei. Then the sections were then mounted with a coverslip using a mounting medium. The following antibodies were used to mark the target molecules: primary antibody, RFP (1:500; 200-301-379, Rockland, Limerick, PA, USA), CD34 (1:500; 14-0341-82, Invitrogen, Carlsbad, CA, USA), cleaved caspase3 (1:300; 9961, Cell Signaling, Danvers, MA, USA), secondary antibody, Cy3-conjugated goat anti-mouse IgG (1:200; S00009-1, Proteintech, Rosemont, IL, USA), FITC-conjugated goat anti-mouse IgG (1:200; S00003-1, Proteintech, USA). Image acquisition was conducted using the Digtal Slide Scan system (AxioScan7, ZEISS, Minato-ku, Tokyo, Japan). All images are processed and analyzed using CaseViewer (version 2.4) and ImageJ (version 1.54f).

### 4.8. Hematoxylin and Eosin (H&E) Staining

Ten-micrometer sections of paraffin-embedded tissue samples were dewaxed with xylene and ethanol, then stained with hematoxylin for 1–5 min for nuclear staining. These sections were differentiated with 1% hydrochloric alcohol for 20 s and counterstained with 1% ammonia water for 30 s. Cytoplasmic staining was performed with eosin for 20 s to 5 min. Finally, the specimens were dehydrated with ethanol and xylene and mounted with a neutral resin for long-term preservation. Image acquisition was conducted using the Digtal Slide Scan system (AxioScan7, ZEISS, Minato-ku, Tokyo, Japan). All images were processed and analyzed using CaseViewer (version 2.4) and ImageJ (version 1.54f).

### 4.9. Flow Cytometry Assays

For single-cell suspension preparation, dorsal skin samples were collected at specified time points and immediately placed in ice-cold PBS. Tissues were minced into 1–2 mm^3^ fragments and subjected to enzymatic digestion in a solution containing 1 mg/mL Collagenase I (171000-17, Gibco, Grand Island, NY, USA), 1 mg/mL Collagenase II (17101-015, Gibco, USA), 2 mg/mL Collagenase IV (17104-019, Gibco, USA), 1 mg/mL Dispase II (04942078001, Roche, South San Francisco, CA, USA), and 125 μg/mL DNase I (10104159001, Roche, USA). The tissue fragments were incubated in the enzyme solution at 37 °C for 60–90 min. The cell suspension was sequentially filtered through 70 μm and 40 μm filters and then centrifuged at 400× *g* for 5 min at 4 °C. Cells were resuspended in RPMI 1640 medium supplemented with 2% FBS, followed by erythrocyte lysis through the addition of three volumes of lysis buffer. For surface marker staining, the cells were incubated with antibodies on ice for 30 min. Then, they were washed twice with staining buffer and resuspended in PBS containing 1% FBS for analysis. Flow cytometry was performed using an Attune NxT flow cytometer (ThermoFisher, Waltham, MA, USA). The following antibodies were used to mark the single-cell suspension: CD34-eFluor660 (mouse, 1:1000; 50-0341-82, eBioscience, San Diego, CA, USA), CD49f-eFluor450 (mouse, 1:1000; 48-0495-82, eBioscience, USA). Data analysis was conducted using the FlowJo software (version 10.8.1). Compensation was performed using single-stained controls, and at least 10,000 live cell events were acquired for each sample. The gating strategy was as follows. First, single cells were selected based on FSC-H vs. FSC-A to exclude cell debris and aggregates. Then, within the single-cell population, 7-AAD-negative cells were selected to exclude dead cells. In the live cell gate, we selected cells that were double-positive for CD34 and CD49f. Simultaneously, tdTomato^+^ cells were selected within the live cell gate. Finally, within the tdTomato+ cell gate, cells that were double-positive for CD34 and CD49f were selected.

### 4.10. RNA Extraction and Quantitative Real-Time Polymerase Chain Reaction (qRT-PCR) Assays

Total RNA was isolated using TRIzol reagent and converted to cDNA using the SuperScript III First Strand Synthesis System (18080051, Invitrogen, Carlsbad, CA, USA) following the manufacturer’s protocol. qRT-PCR was performed using the QuantStudio^TM^ 3 Real-Time PCR System (A28567, ThermoFisher, USA). The PCR reaction was carried out in a 20 μL reaction system using the KAPA SYBR FAST qPCR Master Mix (2×) Universal (KK4601, Roche, USA) and 0.2 μM specific primers. The differences between samples and controls were calculated based on the 2^^-ΔΔCt^ method and shown as fold change normalized to *Actb* expression. The primers used for qRT-PCR were as follows: *Actb*-F (5′-AGAGCCTCGCCTTTGCCGAT-3′), *Actb*-R (5′-CCATCACGCCCTGGTGCCT-3′), *Krt24*-F (5′-GTGGCTCAGCTGTCTGGAAT-3′), *Krt24*-R (5′-ATAGTCACATCCACCCCCGT-3′). All reactions were performed in technical triplicates, and data are presented as mean ± SD of at least three independent biological replicates.

### 4.11. Statistical Analyses

All experimental data were derived from at least three independent biological replicates, with each replicate representing data averaged from three mice. Statistical analyses were performed using Microsoft Excel (version 2021) and GraphPad (version 8.0.1). The data are presented as means ± standard deviation (SD). Unpaired *t*-tests were used to ascertain statistical significance between two groups of every time point. The log-rank test was used to survival analysis between the two groups of mice. A *p*-value of less than 0.05 was considered statistically significant. See figure legends for more information on statistical tests.

## Data Availability

All data supporting the findings in this study are available upon request from the corresponding author.

## Supplementary Materials

The following supporting information can be downloaded at: https://www.mdpi.com/article/10.3390/ijms26073165/s1.

## Abbreviations

*Krt24*: keratin 24: HFSC, hair follicle stem cell; OB, outer bulge; HG, hair germ; ORS, outer root sheath; *Krt5*, keratin 5; *Krt14*, keratin 14; *Krt15*, keratin 15; *Shh*, Sonic hedgehog; *Gli1*, glioma-associated homologue-1; *Inv*, involucrin; *Tyr*, tyrosinase; McSC, melanocyte stem cell; *Postn*, periostin; *Nfatc1*, nuclear factor of activated T cells 1; IRES, internal ribosome entry site; WPRE, woodchuck hepatitis virus posttranscriptional regulatory element; WT, wild-type; TAM, tamoxifen; DT, diphtheria toxin; H&E, hematoxylin and eosin; IR, ionizing radiation; Lgr5, leucine rich repeat containing G protein-coupled receptor 5; BMP, bone morphogenetic protein; FGF, fibroblast growth factor.
