# Supplementary material for: Generation of the Krt24-CreERT2 Mouse Line Targeting Outer Bulge Hair Follicle Cells
Source: Int J Mol Sci. 2025 Mar 29;26(7):3165. doi: 10.3390/ijms26073165 (PMC11988792; doi:10.3390/ijms26073165)
Supplement: Supplementary file 1 [file ijms-26-03165-s001.zip › ijms-3535984-supplementary.pdf]

Supplementary Figures

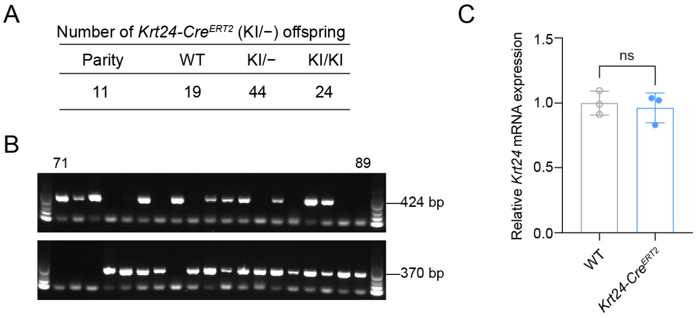

Number of *Krt24-Cre^{ERT2}* (KI/−) offspring			
Parity	WT	KI/−	KI/KI
11	19	44	24

Supplementary Figure S1. Generation of the *Krt24-Cre^{ERT2}* Mouse Line Targeting Outer Bulge Hair Follicle Cells.

(A) Breeding data for *Krt24-Cre^{ERT2}* (KI/−) mice are shown, including litter size and genotype distribution. The average litter size was 7.9 pups, with homozygous (KI/KI) offspring accounting for approximately 28% of the progeny. (B) PCR products of primers F3/R3 and F4/R4 can detect wild-type and knock-in allele, respectively. The 370-bp product from the primer F4/R4 was observed in KI/− and KI/KI mice. The 424-bp product from the primer F3/R3 was observed in WT and KI/−mice, but not in KI/KI mice. The band labeled “M” represents the DNA marker. (C) qRT-PCR experiments were performed to determine *Krt24* mRNA expression levels in dorsal skin of WT mice and *Krt24-Cre^{ERT2}* mice. Quantitative comparisons revealed comparable transcript abundance of *Krt24* in epidermal tissues between homozygous *Krt24-Cre^{ERT2}* mice and WT. KI, knock in; WT, wild-type.

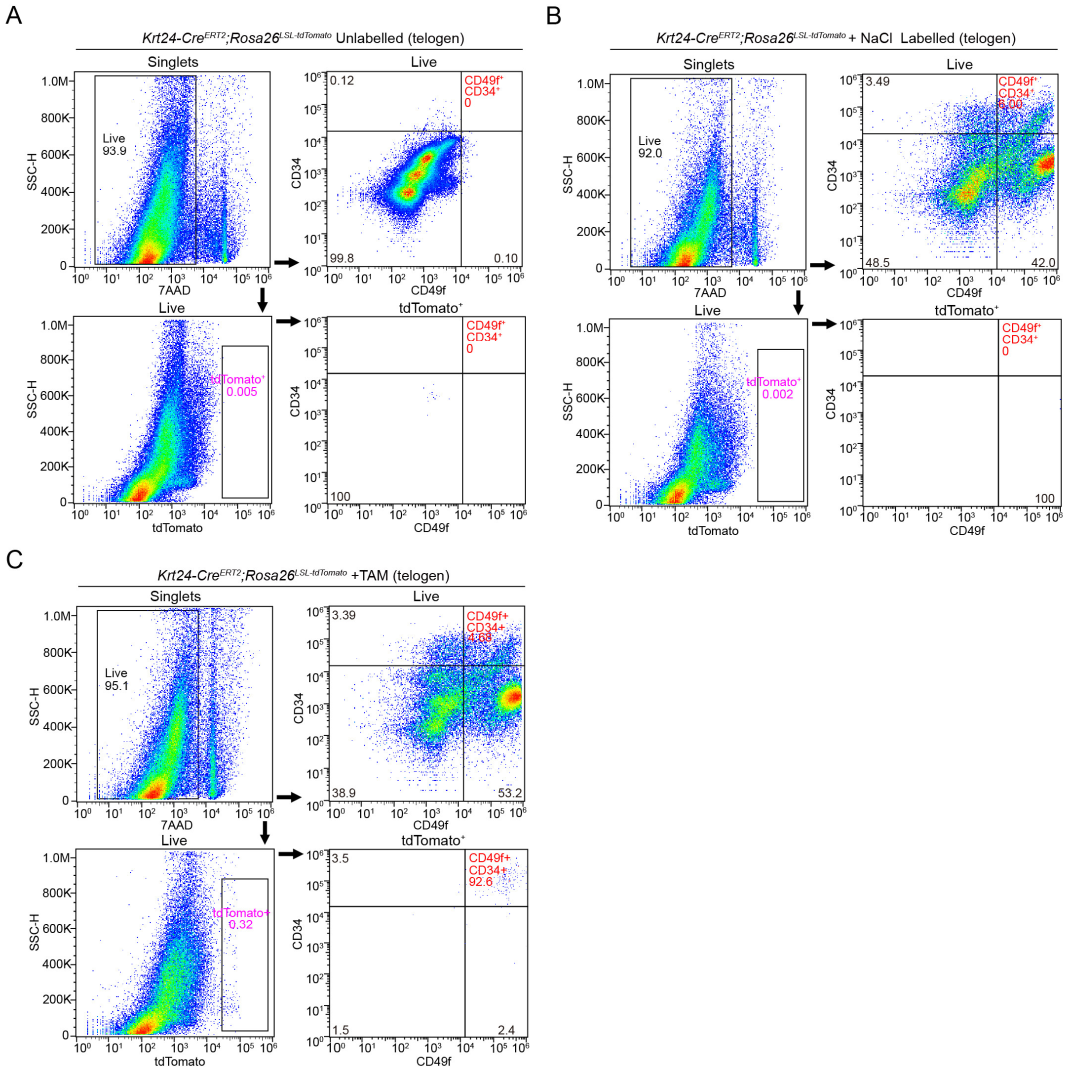

Supplementary Figure S2. TdTomato-Labeled *Krt24*⁺ Cells Belong to Outer Bulge Hair Follicle Stem Cells.

(A) Flow cytometric analysis of dissociated cells from the dorsal skins of *Krt24-Cre^{ERT2}* mice after TAM treatment, in which the cells were not labeled with antibodies, serving as a blank control. *n* = 3 mice. (B) Flow cytometric analysis of dissociated cells from the dorsal skins of *Krt24-Cre^{ERT2};Rosa26$^{LSL\text{-}tdTomato}$* mice after 0.9% NaCl treatment, in which the cells were labeled with indicated antibodies. *n* = 3 mice. (C) Flow cytometry analyses of dissociated cells from dorsal skins of *Krt24-Cre^{ERT2};Rosa26$^{LSL\text{-}tdTomato}$* mice after TAM treatment, in which the cells were labeled with indicated antibodies. The gating strategy was as follows: First, single cells were selected based on FSC-H vs. FSC-A to exclude cell debris and aggregates. Then,

within the single-cell population, 7-AAD-negative cells were selected to exclude dead cells. In the live cell gate, we selected cells that were double-positive for CD34 and CD49f. Simultaneously, $tdTomato^{+}$ cells were selected within the live cell gate. Finally, within the tdTomato+ cell gate, cells that were double-positive for CD34 and CD49f were selected. *n* = 3 mice. TAM: tamoxifen.
